# Anterior segment optical coherence tomography imaging and ocular biometry in cataract patients with open angle glaucoma comorbidity

**DOI:** 10.1186/s12886-021-01874-x

**Published:** 2021-03-08

**Authors:** Giedre Pakuliene, Kirilas Zimarinas, Irena Nedzelskiene, Brent Siesky, Loreta Kuzmiene, Alon Harris, Ingrida Januleviciene

**Affiliations:** 1 Ophthalmology Department, Lithuanian University of Health Sciences, Eiveniu g. 2, 50161 Kaunas, Lithuania; 2 Biostatistician, Odontology faculty, Department of Dental and Oral Pathology, Lithuanian University of Health Sciences, Kaunas, Lithuania; 3 Icahn School of Medicine at Mount Sinai, New York, NY USA

**Keywords:** Anterior chamber, Cataract, Glaucoma, open-angle, Narrow-angle, Lens-crystalline

## Abstract

**Background:**

Anterior chamber angle anatomy in perspective of ocular biometry may be the key element to intraocular pressure (IOP) reduction, especially in glaucoma patients. We aim to investigate anterior chamber angle and biometrical data prior to cataract surgery in patients with and without glaucoma comorbidity.

**Materials and methods:**

This prospective comparative case-control study included 62 subjects (38 with cataract only and 24 with cataract and glaucoma). A full ophthalmic examination including, Goldmann applanation tonometry, anterior chamber swept source optical coherence tomography (DRI OCT Triton plus (Ver.10.13)) and swept source optical biometry (IOL Master 700 v1.7) was performed on all participants.

**Results:**

We found that ocular biometry parameters and anterior chamber parameters were not significantly different among groups. However, when we added cut-off values for narrow angles, we found that glaucoma group tended to have more narrow angles than control group. IOP was higher in glaucoma group despite all glaucoma patients having medically controlled IOP. In all subjects, anterior chamber parameters correlated well with lens position (LP), but less with relative lens position, while LP cut-off value of 5.1 mm could be used for predicting narrow anterior chamber angle parameters.

**Conclusions:**

Cataract patients tend to develop narrow anterior chamber angles. Anterior chamber angle parameters have a positive moderate to strong relationship with lens position. LP may be used predicting narrow angles.

## Background

Cataract and glaucoma are both comorbid age related diseases, which alter normal ocular anatomy [1–4]. Specifically, lens opacification induces thickening of lens and shallowing of anterior chamber depth and these changes are even more pronounced with age [5]. Several studies refer to the reduction of intraocular pressure (IOP) after phacoemulsification; however the results vary among different authors [6–10]. Primary angle closure glaucoma (ACG) patients and primary angle closure suspects (PACS) have greater IOP reduction than open angle glaucoma (OAG) patients or otherwise healthy individuals [6, 8]. Even though the mechanisms behind IOP reduction after phacoemulsification are still debated, these results suggest that angle anatomy is an important landmark in IOP reduction after cataract surgery.

Varma et al. found, that almost 1 in 11 patients, referred as “open angle glaucoma”, was in fact found to have closed angles [11]. In another study, Varma et al. found, that more than 12% of all cataract surgery referrals, referred either by an ophthalmologist or an optometrist, have closed angles or were PACS [12].

Anterior segment optical coherence tomography (AS-OCT) is a reliable anterior chamber angle evaluation method [13]. The modality allows well quantifiable and highly repeatable measurements of anterior chamber angle parameters [13]. Siak et al. found that even though the IOP reduction after phacoemulsification was similar between ACG and OAG groups, the AS-OCT anterior chamber angle was more open in OAG than in ACG group [14]. However, they did not evaluate the lens position, which may be an important consideration.

In this analysis we investigated AS-OCT imaging and anterior chamber angle parameters in cataract patients with or without previously diagnosed OAG in perspective of ocular biometry and lens position results. The focus of our study included cataract surgery patients, referred by an ophthalmologist, with no suspicion of closed or narrow anterior chamber angle. To the best of our knowledge, this is the first study evaluating AS-OCT anterior chamber angle parameters in cataract patients with or without OAG in perspective of biometry results and lens position preoperatively.

## Methods

This prospective comparative case-control study was carried out in the Lithuanian University of Health Sciences, Ophthalmology Department in 2018–2019. All procedures were approved by the Kaunas Regional Biomedical Research Ethics Committee and all subjects signed an informed consent prior to participation.

Inclusion criteria: patients, who were scheduled for cataract surgery and having open angle (Shaffer III – IV gonioscopically), as referred by district ophthalmologist. The study group consisted of cataract patients over 18 years with diagnosed and medically controlled OAG, while the control group consisted of cataract patients over 18 years with no other eye disease.

Exclusion criteria: subjects with vision < 6/24 (Snellen chart), glaucoma suspects, patients with obvious lens subluxation or lens swelling (to exclude lens induced glaucoma), suspected angle closure a priori. We excluded OAG patients with IOP > 21 mmHg, and additionally medications for IOP reduction were recorded for the OAG patients.

All of the subjects received a full ophthalmic examination, including IOP via Goldmann applanation tonometry, AS-OCT (DRI OCT Triton plus (Ver.10.13)) for anterior chamber angle tomograms (measuring angle opening distance at 500 μm from scleral spur (AOD_500_), trabecular iris space at 500 μm and 750 μm from scleral spur (TISA_500_ and TISA_750_) and swept source optical biometry (IOL Master 700 v1.7) for ocular biometry (axial length (AL), anterior chamber depth (ACD), lens thickness (LT), central corneal thickness (CCT), spherical equivalence (SE), white-to-white corneal diameter (WTW). ACD was measured from epithelium. IOP was measured after AS-OCT and biometry, to avoid artefacts. AS-OCT imaging was performed under dark room conditions without pupil dilation and AS-OCT images were processed and evaluated using Fiji program package [15]. The anterior chamber angles were manually evaluated by two independent observers (G.P. and K.Z.) in blinded manner. The intraobserver repeatability and interobserver agreement were excellent (Intraclass correlation coefficient (ICC) ≥0.9). All of the AS-OCT scans were performed using “Line 6 mm” option with external fixation. AS-OCT scans were performed at the 3, 6, 9, 12 o’clock position of anterior angle structures. We chose to proceed with 3 and 9 o’clock positions, because had a lot of scleral artefacts, and not all tomograms were appropriate for evaluation. This could be due to age of the patients.

**Fig. 1:**
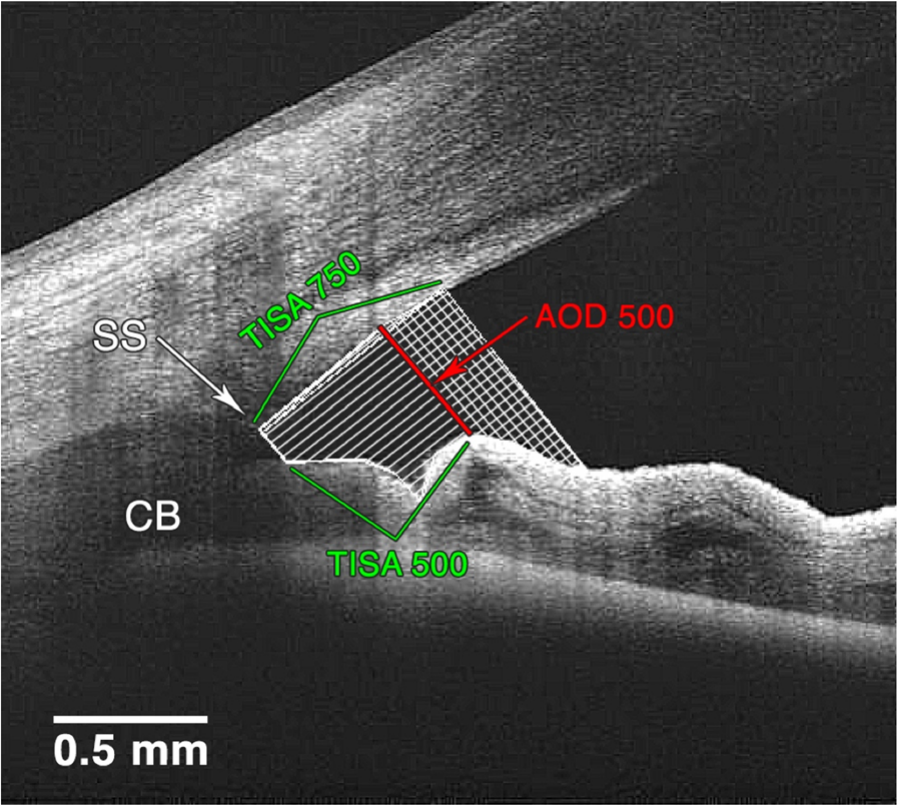
AOD_500_, TISA_500_ and TISA_750_ measurements on AS-OCT image of anterior chamber angle (as described in the text)

The measurements were made as follows (Fig. 1):

1. Angle opening distance (AOD_500_) – the distance from the point on the cornea (which was 500 μm from the scleral spur) to a perpendicular point on the iris (as described by Pavlin

et al. [16]). Angle opening distance at 750 μm (AOD_750_) was used in TISA_750_ measurement.

2. Trabecular iris space area (TISA_500_ and TISA_750_) [17]– the area with defining boundaries of:

1. Anterior wall – AOD_500_ or AOD_750_ respectively;
2. Posterior wall – a line, beginning at scleral spur, drawn perpendicularly from the inner scleral wall to the iris;
3. Superior wall – corneoscleral surface between anterior and posterior walls;
4. Inferior wall – iris surface between anterior and posterior walls.

The TISA_500_ and TISA_750_ were measured as described by Radhakrishnan et al. [17].

Narrow anterior chamber angle is considered < 20° and < 10° upon gonioscopy according to Shaffer classification, with probable and possible angle closure respectively [18]. Individuals with anterior chamber angle < 20° were considered PACS, despite not having any glaucomatous changes [18]. According to Radhakrishnan et al., the cut-off value for indicating occludable angles (≤10°) in AS-OCT were 191 μm for AOD_500_, 0.11mm^2^ for TISA_500_ and 0.17mm^2^ for TISA_750_ [17]. We chose the same cut-off values to reevaluate the data. There were several studies, which suggested different cut-off values for occludable anterior chamber angles in AS-OCT [17, 19–21]. We chose previously mentioned cut-off values, because we found similar results in our pilot study (mean and median).

Swept source optical biometry was performed in light room conditions without pupil dilation. The measurements collected were:

1. Axial length (AL) (mm);
2. Anterior chamber depth (ACD) (mm);
3. Lens thickness (LT) (mm);
4. Spherical equivalent (SE) (D);
5. Horizontal corneal diameter (white to white WTW) (mm);
6. Central corneal thickness (CCT) (μm).

LP and RLP were derivative values from AL, ACD and LT. LP was found adding ACD and ½ LT. RLP was found LP dividing by AL [22].

In order to compare AOD_500_, the calculated sample size to provide 80% power to detect a difference of 50 μm with SD of 58 μm [17] between control and study patients was at least 22 in each group assuming two-sided tests and a 95% significance level​.

### Primary and secondary outcomes

The main outcomes of our study included comparing anterior chamber angle and ocular biometrical measurements between cataract patients with or without OAG. The secondary outcomes were the assignation to whether the anterior chamber angle was open or narrow, and finding correlations between anterior chamber angle parameters and lens position (LP) and relative lens position (RLP).

Statistical analysis was performed using IBM SPSS Statistics for Windows, Version 23.0. (Armonk, NY: IBM Corp) program package. Appropriate statistical test was chosen to evaluate results. Kolmogorov-Smirnov test was used to determine distribution of the data. Student’s t test was used for normally distributed independent samples. Quantitative data was presented as Mean (SD). Mann-Whitney U test was used for non-parametric independent samples; the data was presented as Median (IQR). Pearson’s Correlation Coefficient (PCC) was used for correlations. In order to assess minimally false negative and minimally false positive results with greatest accuracy, the method of ROC (Receiver Operating Characteristics) curve was used. Statistical significance was set at *p* < 0.05.

## Results

Sixty-two subjects were included in the study with a control group of 38 (61.3%) subjects and the glaucoma group included 24 (38.7%) subjects. All of the subjects were of Caucasian ethnicity. Gender ratio and age were similar in both groups (Table 1). Even though OAG group subjects had medically controlled glaucoma, their IOP was statistically significantly higher than the control group.

**Table 1:** Comparison of Demographic Data

	Control	OAG	***p***
Number of eyes	38	24	–
Age (mean (SD) y	74.1 (6.6)	74.7 (8.5)	0.768
Female/male (%)	68.4/31.6	75.0/25.0	0.58
IOP (median, (IQR))	14.97 (13.0–17.0)	16.83 (17.0–18.5)	**0.014**

*IOP* Intraocular pressure, *IQR* Interquartile range. Gender ratio and age are similar in both groups. OAG group had higher IOP

### Ocular biometry

Ocular biometry measurements were similar in both groups, except for CCT, which was statistically significantly lower in OAG group (Table 2).

**Table 2:** Comparison of Ocular Biometrical Data

Mean (SD)	Control	OAG	***p***
AL, mm	23.2 (1.27)	23.0 (1.27)	0.29
ACD, mm	3.0 (0.38)	2.9 (0.34)	0.56
LT, mm	4.6 (0.40)	4.7 (0.33)	0.26
CCT, mm	567.5 (38.3)	534.0 (28.4)	**0.001**
SE,D	43.7 (1.24)	44.2 (1.7)	0.20
WTW, mm	11.7 (0.41)	11.6 (0.42)	0.38
LP	5.3 (0.31)	5.3 (0.29)	0.997
RLP	0.23 (0.012)	0.23 (0.016)	0.488

*AL* Axial length, *ACD* Anterior chamber depth, *LT* Lens thickness, *CCT* Central corneal thickness, *SE* Spherical equivalent, *WTW* White to white – horizontal corneal diameter, *SEM* Standard error of mean. AL, ACD, LT, SE and WTW were similar among the groups

We found moderate negative correlation between ACD and LT in both control (*r* = − 0.595, *p* < 0.001) and glaucoma groups (*r* = − 0.521, *p* = 0.009). In control group ACD showed moderate positive correlation with AL (*r* = 0.559, *p* < 0.001), however, no correlation between ACD and AL was found in glaucoma group (*p* = 0.318) (PCC).

A moderate negative correlation was seen between AL and SE in both control group (*r* = − 0.448, *p* = 0.005) and glaucoma groups (*r* = − 0.463, *p* = 0.026). CCT did not correlate with AL, ACD or LT in neither of the groups (*p* > 0.05) (PCC).

### Anterior chamber angle

Anterior chamber angle measurements were similar between the two groups (Table 3).

**Table 3:** Comparison of anterior chamber angle measurements

Mean (SD) (mm)	Control	OAG	p
AOD_500_ nasal	0.41 (0.20)	0.37 (0.18)	0.356
AOD_500_ temporal	0.41 (0.18)	0.39 (0.15)	0.580
TISA_500_ nasal	0.16 (0.08)	0.15 (0.07)	0.440
TISA_500_ temporal	0.16 (0.06)	0.15 (0.07)	0.617
TISA_750_ nasal	0.27 (0.12)	0.24 (0.12)	0.752
TISA_750_ temporal	0.28 (0.10)	0.24 (0.11)	0.692

*AOD*_*500*_ Anterior angle opening distance at 500 μm from scleral spur. *TISA*_*500*_ *and TISA*_*750*_ Trabecular iris space area at 500 μm and 750 μm from scleral spur respectively. AOD_500_, TISA_500_ and TISA_750_ were similar nasally and temporally among groups

We chose the previously mentioned cut-off values to reevaluate the data [17]. The percentage of narrow angles in cataract and cataract with OAG groups according to AOD_500_ were approximately 11.1 and 21.7% (*p* > 0.05), according to TISA_500_ were 25.0 and 30.4% (*p* > 0.05), according to TISA_750_ were 22.2 and 30.4% (*p* > 0.05). The percentages of narrow angles were similar in both groups nasally and temporally, with a tendency of slightly higher percentage in OAG group.

The number of different hypotensive substance used by OAG patients did not correlate with anterior chamber angle parameters, nor ACD (*p* > 0.05, PCC).

### LP, RLP and AOD_500_

**Fig. 2:**
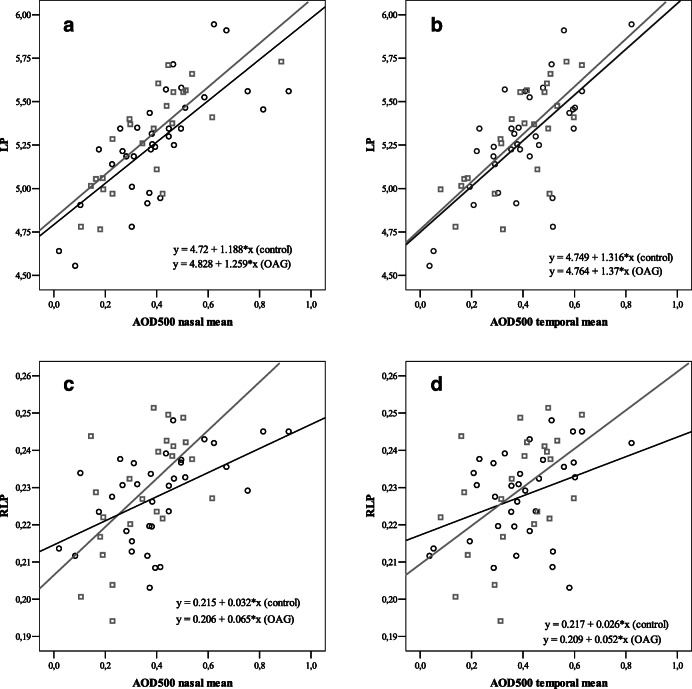
Correlation graphs of AOD_500_ temporally and nasally with LP and RLP. Correlations between LP, RLP and AOD_500_. LP and AOD_500_ correlations were strong in control and glaucoma groups (**a-b**). AOD_500_ correlates with RLP slightly less than with LP (**c-d**)

LP showed strong positive correlation with AOD_500_ nasally (*r* = 0.733, *p* < 0.001) and temporally (*r* = 0.690, *p* < 0.001) in control group and accordingly nasally (*r* = 0.777, *p* < 0.001) and temporally (*r* = 0.727, *p* < 0.001) in OAG group. RLP showed moderate positive correlation with AOD_500_ nasally (*r* = 0.524, *p* = 0.001) and weak positive correlation temporally (*r* = 0.362, *p* = 0.034) in control group. In OAG group, RLP showed moderate positive correlation with AOD_500_ nasally (*r* = 0.587, *p* = 0.036), and temporally (*r* = 0.493, *p* = 0.017) (Fig*.* 2).

### LP, RLP and TISA_500_

**Fig. 3:**
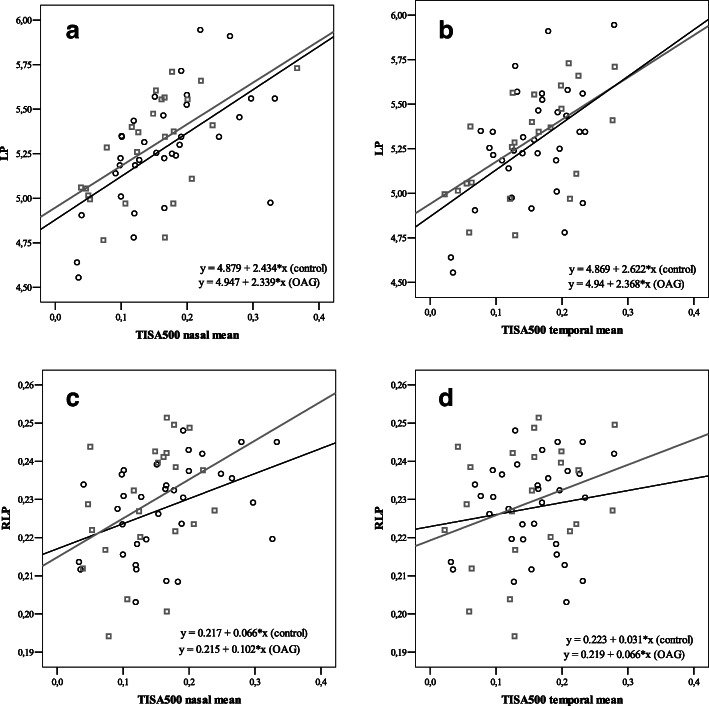
Correlations between TISA_500_ and LP were significant in control and glaucoma groups (**a-b**). TISA_500_ and RLP showed moderate correlation temporally and did not correlate nasally in control group. TISA_500_ and RLP did not correlate in glaucoma group (**c-d**)

LP showed moderate positive correlation with TISA_500_ nasally (*r* = 0.593, *p* < 0.001), and temporally (*r* = 0.489, *p* = 0.003) in control group while strong positive correlation nasally (*r* = 0.597, *p* = 0.002) and moderate positive correlation temporally (*r* = 0.591, *p* = 0.003) in OAG group. RLP showed moderate positive correlation with TISA_500_ nasally (*r* = 0.420, *p* = 0.013), however did not show any correlation with TISA_500_ temporally (*p* = 0.412) in control group. RLP did not show any correlation with TISA_500_ neither nasally, nor temporally in OAG group (*p* > 0.05) (PCC) (Fig*.* 3).

### LP, RLP and TISA_750_

**Fig. 4:**
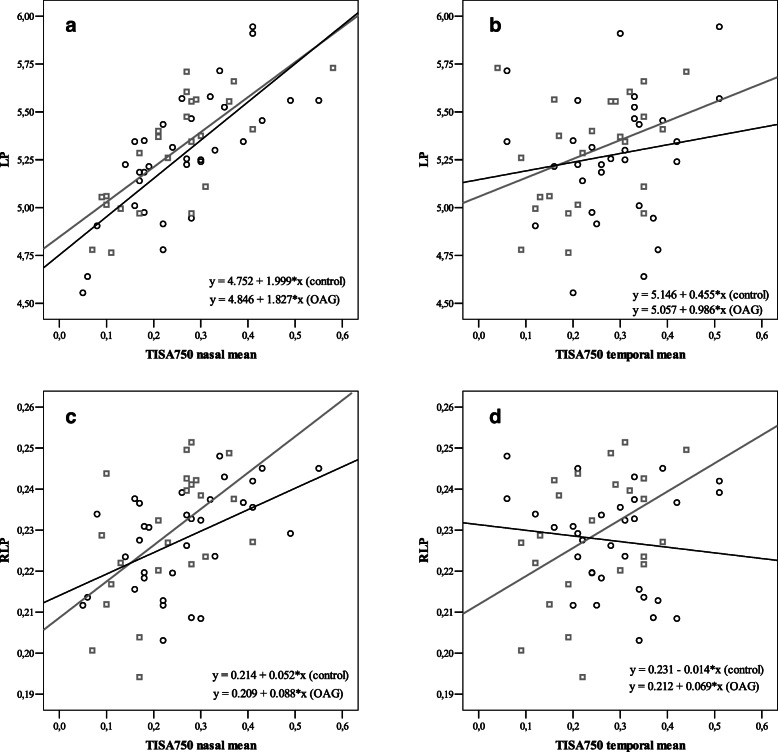
Correlations between TISA_750_, LP and RLP. LP showed strong correlation with TISA_750_ in control and glaucoma groups (**a-b**). RLP showed moderate correlation with TISA_750_ nasally, but did not correlate temporally in control group. RLP showed moderate correlation with TISA_750_ nasally and temporally (**c-d**)

LP showed strong positive correlation with TISA_750_ nasally (*r* = 0.738, *p* < 0.001), but not temporally (*p* > 0.05) in control group. In glaucoma group, LP showed strong positive correlation with TISA_750_ nasally (*r* = 0.747, *p* < 0.001), but no correlation temporally (*p* > 005). RLP showed moderate positive correlation with TISA_750_ nasally (*r* = 0.506, *p* = 0.003), however did not correlate with temporal side (*p* > 0.05) in control group. RLP showed moderate positive correlation with TISA_750_ nasally (*r* = 0.536, *p* = 0.008) and temporally (*r* = 0.436, *p* = 0.048) in OAG group (PCC) (Fig*.* 4).

**Fig. 5:**
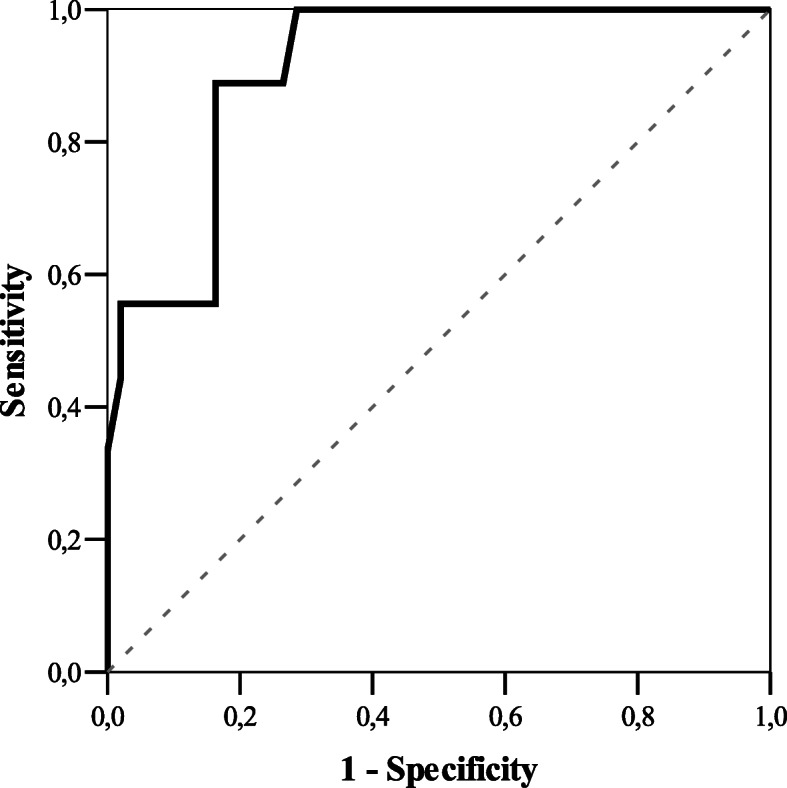
Area Under the Curve 91.0%, sensitivity 88.9%, specificity 83.5%. ROC analysis for cut-off LP value prognosing AOD_500_ (at cut-off of 191 μm)

The LP and anterior chamber angle parameters were not statistically different among groups, so we proceeded following calculation combining both groups. ROC analysis the cut-off value of LP 5.1 (mm), considering AOD_500_ cut-off value (191 *μ*m) [17]. If LP was *>* 5.1 (mm), AOD_500_ was *<* 191 *μ*m in 2.4% of cases (*n* = 1). If LP was *<* 5.1 mm, AOD_500_ was *<* 191 *μ*m in 50% of cases (*n* = 8) (*p* < 0.001) (Fig*.* 5).

## Discussion

Our study aimed to evaluate anterior chamber angle parameters and biometrical ocular data in individuals with cataract and with or without OAG. We did not find any statistically significant differences between ocular biometry parameters in control and OAG groups (regarding AL, ACD, LT, SE, WTW and the derivate values LP and RLP). The OAG group had slightly narrower anterior chamber angles than control group, however the difference was statistically insignificant.

The CCT was lower in the OAG group and this result was in agreement with previous studies [23]. We found that mean LT was 4.6 mm in control group and 4.7 mm in OAG group which were similar to results previously reported in cataract patients without glaucoma in studies by Shammas et al. (mean LT 4.6 mm) [5], Jivrajka et al. (mean LT 4.93 mm) [24] and Hoffer et al. (mean LT 4.63 mm) [25]. Shammas et al. also found, that the most increase of LT attributed to anterior cortex space [5].

Wang et al. found that non-glaucomatous white individuals had more posteriorly positioned lenses, than Asians, African Americans and Hispanics [26]. However, in our study we found, that LP preoperatively was more anteriorly positioned in both groups than in white individuals’ sample in Wang et al. study [26]. The difference could occur due to a slightly older sample in our study. The LP and RLP in our study were similar in control and OAG groups. According to our results, cataract patients with and without OAG, showed no differences in LP prior to cataract surgery. Importantly, we found the a cut-off LP value of 5.1 mm was predictive of whether the patient had a narrow angle. In the future, this measurement could be used predicting narrow angles with ocular biometry, alongside gonioscopy.

Mean AOD_500_, TISA_500_ and TISA_750_ were statistically similar in control and OAG groups. This suggests that individuals, who developed cataract, had in general similar anterior chamber angle parameters, despite having or not having glaucoma. Using the same cut-off values as Radhakrishnan et al., we also found parameters were slightly different between cataract vs. cataract and glaucoma groups [17]. Although the differences between groups were not statistically significant, we found that OAG group tended to have narrow angles slightly more often than control group. In addition to that, we found, that IOP was statistically significantly higher in OAG than in control group, despite the fact that OAG patients had sufficiently medically controlled IOP.

Our study showed a slightly higher percentage of narrow angles in both control and OAG groups, than Varma et al. [12]. They found that 12.9% of cataract referrals had PACS/angle closure, however it is important to note that our study patients were much older than in Varma et al. study [12]. This difference in age could explain a higher percentage of PACS/angle closure. Additionally, Varma et al. used gonioscopy to determine narrow angles, while we used AS-OCT; and the different evaluation method could lead to different results [12]. Varma et al. suggested that this percentage of undiagnosed narrow angles in their study could be because gonioscopy was underperformed and narrow angles were missed [12]. We suggest that since anterior chamber is a dynamic structure, in some cases the overall view may alter over time and differ from previous examinations, especially in cataract cases. However, the Varma et al. study did not specify whether glaucoma had been already diagnosed in the study group [12]. The undetected narrow angles for individuals with glaucoma may have impact on IOP management.

The literature suggests that LT has an inverse relationship with AL and ACD [27]. In our analysis we found that anterior chamber angle parameters (AOD_500_, TISA_500_) had a strong positive correlation with LP (which was calculated using ACD and LT). We also found, that anterior chamber angle parameters depended on ACD and LT (LP) more than ACD, LT and AL (RLP). This could be explained by increase in lens thickness during cataract formation in mainly anterior cortical space, which influenced anterior chamber angle parameters and due to this AL influence was less significant [5].

Radhakishnan et al. in AOD_500_, TISA_500_ and TISA_750_, showed slightly larger parameters measuring temporal quadrant comparing to nasal quadrant. This was neither emphasized as statistically significant difference, nor of overall importance in the original article [17]. Our measurements between nasal and temporal quadrants were slightly, yet not statistically different. When we computed correlations between angle measurements and RLP, we noticed that in control group correlations were weaker or absent in the temporal quadrant, but remained at least moderate in nasal quadrant. In glaucoma group, AOD_500_ and TISA_750_ maintained strong or moderate correlation with RLP in both quadrants, but TISA_500_ lost it. This may be partially explained by the lens tilt, as previous studies suggest lens tilt to be up to 5 degrees with outward nasal orientation with mirror symmetry in both eyes [28, 29].

We also found, that if LP were below cut-off value (< 5.1), it was more likely, that AOD_500_ was < 191 μm, which falls into a “narrow angle” category.

Our study had several advantages as compared to similar studies. We used objective evaluation of ocular biometric parameters, which were more accurate and were possible even through dense nucleus [30–32]. Another advantage was, that we used AS-OCT anterior chamber angle measurements in accurate close up images, which did not require contact and our measurements were highly repeatable.

Along with advantages our study also had several limitations to acknowledge. First, our study did not differentiate, which part of the lens was most affected by the cataract – nuclear, subcapsular or cortical, as did Shammas et al. [5]. Instead, we provided objective measurement (LP and RLP), which change, if the lens thickens in cortex, nucleus, subcapsular masses, or in combination of lens parts. We also did not differentiate OAG group by glaucoma medications (only by number of different substances), which may influence results. Additionally, it is worth to mention, that all of the measurements, presented in our study, were derivative and not direct, yet remaining objective.

## Conclusion

Ocular biometry data and anterior chamber angles were similar among cataract patients with and without glaucoma. CCT was lower in glaucomatous subjects. Patients with cataract, despite having or not having glaucoma, tended to develop narrow angles. Patients with cataract and glaucoma had higher preoperative IOP, comparing to control group, even though they had medically controlled glaucoma. AS-OCT helped to obtain useful quantifiable information of anterior chamber angle anatomy. LP cut-off value of 5.1 mm was found to be able to differentiate between open and narrow angle (AOD_500_) with high sensitivity and specificity. IOL-Master700 was an effective tool to evaluate ocular biometry parameters even through a dense nucleus. These approaches could lay a new perspective to future studies and future studies with different age groups and different stages of glaucoma are needed to evaluate possible influence on IOP change due to narrowing of angles during cataract formation. In addition, LP as a predictor of narrow anterior chamber angle may be important to evaluate in larger longitudinal studies.

## Data Availability

The data used in this study is available at reasonable request to corresponding author.

## Abbreviations

AS-OCT: Anterior segment optical coherence tomography
ACG: Angle closure glaucoma
ACD: Anterior chamber depth
AL: Axial length
AOD_500_: Angle opening distance
CCT: Central corneal thickness
IOP: Intraocular pressure
LT: Lens thickness
LP: Lens position
OAG: Open angle glaucoma
PACS: Primary angle closure suspects
RLP: Relative lens position
SE: Spherical equivalent
TISA_500_ and TISA_750_: Trabecular iris space area 500μm and 750μm from scleral spur respectively
WTW: Horizontal corneal diameter (white to white)
